# Pleomorphic adenoma of hard palate: Review of updated literature and “case report”

**DOI:** 10.1097/MD.0000000000039529

**Published:** 2024-09-06

**Authors:** Lujain AlSahman, Osama Alghamdi, Hamad Albagieh, Ali AlRefai, Roba AlSahman, Shatha Alnafea

**Affiliations:** a Department of Oral Medicine and Diagnostic Sciences, College of Dentistry, King Saud University, Riyadh, Saudi Arabia; b Department of Oral and Maxillofacial Surgery, College of Dentistry, King Saud University, Riyadh, Saudi Arabia; c Faculty of Dentistry, Royal College of Surgeons, Dublin, Ireland.

**Keywords:** encapsulated lesion, hard palate, pleomorphic adenoma, salivary gland tumor

## Abstract

**Rational:**

Pleomorphic adenoma (PA) is a rare benign tumor mainly affecting the major salivary glands, known for its diverse histological appearances that can mimic malignancies. When it occurs in the hard palate it present diagnostic and management challenges compared to other sites due to the anatomical location and potential proximity to critical structures. This case reports a rare presentation PA starting as an ulcer, alongside a review of rare cases of PA reported in last 5 years. We aim to highlight clinical challenges and emphasize the need for awareness in diagnosis of this diverse entity amongst the clinicians before reaching a definitive conclusion.

**Patient concerns:**

A 41-year-old female reported an asymptomatic large swelling on the right side of the posterior palatal region. Clinical diagnosis revealed a firm, rubbery, and non-tender swelling of approximately 4 cm × 4 cm diameter. A triangular incisional biopsy was performed to confirm the diagnosis.

**Diagnosis:**

The histopathological evaluation confirmed the presence of a PA with a well-encapsulated and compressed salivary gland. A wide surgical dissection was made to remove the entire encapsulated tumor mass, including the mucoperiosteum and eroded bone of the palate. The borderline of the tumor was carefully identified in the surrounding healthy tissue. The hemostasis was obtained using a simple interrupted suture.

**Lesson:**

The diagnosis of PA is difficult as it usually involves extensive squamous and mucous metaplasia, confusing it with malignant disorders. Histopathological and clinical examinations are important for differentiating this lesion from other tumors. Complete surgical excision is reported as the first line of treatment.

## 1. Introduction

Salivary gland tumors are morphologically and clinically heterogeneous, making diagnosis and management difficult for oral pathologists and surgeons. Pleomorphic adenoma (PA), a mixed tumor, is a most common salivary gland tumor of benign origin.^[1]^ Histologically, this tumor is characterized as epithelial and mesenchymal origin, predominantly affecting major salivary glands. Approximately 75% of this tumor is reported in the parotid gland, followed by the submandibular gland (15–17%) and the minor nasal and sublingual gland (10–12%).^[2]^ This neoplasm is rarely reported in the minor glands of the larynx, lips, floor of the mouth, and trachea (5–7%).^[3]^ The palatal mucosa is the most common intraoral site, followed by the upper lip and buccal mucosa. Rarely, PAs have been reported in soft tissues, lymph nodes, lacrimal glands, upper and lower limbs, breast, axilla, ear, and mediastinum.^[4]^ PAs can occur synchronously and asynchronously with other oral lesions and tumors. The neoplasm typically presents with mild symptoms, thus rendering it difficult to make an initial clinical diagnosis during the prehospital stage. Although PA may appear benign, its biological behavior and prognosis indicate a low-grade malignancy, necessitating immediate treatment wherever diagnosed.

It is comprehended that this tumor can be diagnosed and surgically treated using computed tomography, magnetic resonance imaging, ultrasound, sialography, and partial, subtotal, and ultimately total parotidectomy.^[5]^ Histopathological analysis forms the foundation for the definitive diagnosis of PA.^[6]^ The origin of the tumor (tumor phenotype) can be identified using morphological methods in addition to identifying whether the tumor is benign or malignant. There are various morphological classifications of PA in the literature depending upon their nature of origin.^[7]^ However, despite various treatment modalities, diagnosis methods, etiology, and clinical representation of patients with PA, diagnosis and treatment measures are still debatable. Hence in the present case report, authors represented and managed the case with PA of hard palate along with a pertinent literature review on rare cases. Written informed consent was obtained from the patient for the use of her clinical, imaging, and other relevant data in the manuscript for the purpose of publication.

## 2. Case presentation

A 41-year-old female was referred to the Department of Oral and Maxillofacial Surgery, College of Dentistry, XX University, by the Department of Oral Diagnosis, regarding an asymptomatic mass on her palatal mucosa adjacent to her right maxillary molar. The patient first recorded the swelling 2 years ago, which started as an ulcer and gradually increased in size. The patient recalled a watery discharge from the swelling a few weeks ago. No relevant medical and dental history was elicited. The patient was a nonsmoker and nonalcoholic. On facial examination, no facial asymmetry or tenderness was recorded.

### 2.1. Clinical examination

**Figure 1.**
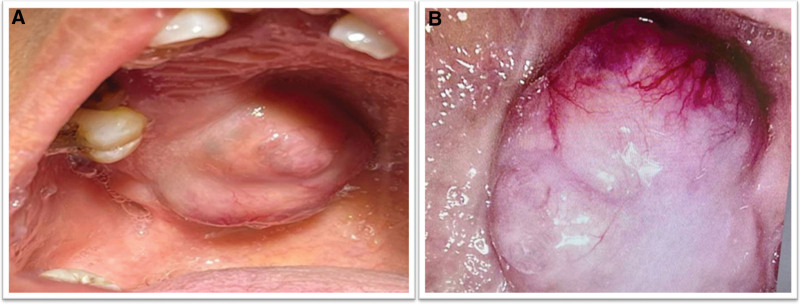
(A) Intraoral view of the lesion; (B) nodular and pink appearance of the lesion.

**Figure 2.**
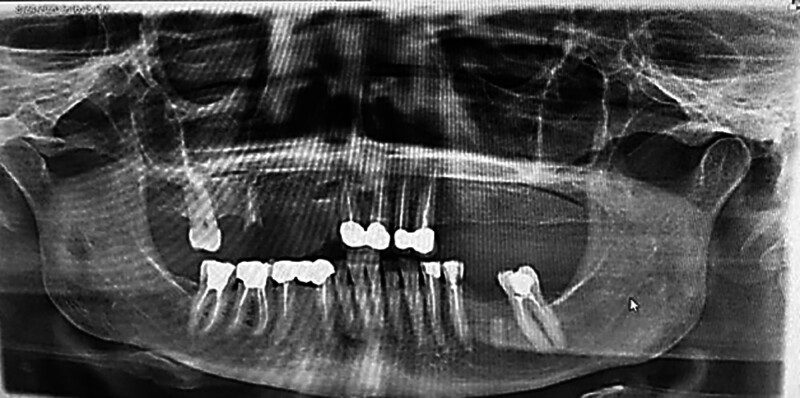
Radiographic representation of the lesion.

On examination, a firm, rubbery, and non-tender swelling of approximately 4 cm × 4 cm diameter was recorded. The swelling was present in deep palatal mucosa, covering the root of the first molar on the right side and crossing the palatal midline (Fig. 1A). The underlying mucosa was pink, smooth, and warm on palpation (Fig 1B). Orthopantomogram reveals no underlining bony defect (Fig. 2). All the vital signs of the patient were within normal range. A provisional diagnosis of an inflammatory mass, PA, and neoplasm was made.

### 2.2. Incisional biopsy

**Figure 3.**
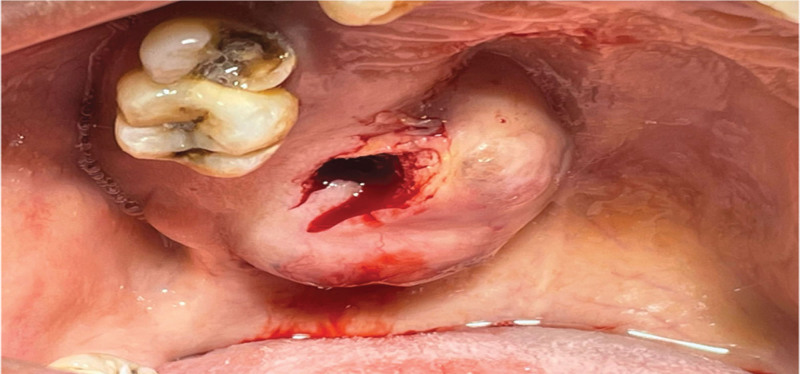
Triangular incisional biopsy.

After explaining the patient’s differential diagnosis of the lesion, informed consent was obtained for the biopsy. The patient was kept under observation, and an incisional biopsy was planned. Fine needle aspiration was not planned due to the firm nature of the lesion. A triangular incisional biopsy with a #15 blade was made (Fig. 3), and the sample was collected in 3 containers (A, B, and C). The lesion’s hemostasis was obtained using silk-interrupted suture (Fig. 3).

### 2.3. Macroscopic description and findings

Obtained specimens were preserved in the formalin solution. Specimens were placed in 3 containers (A) consisting of soft tissue measuring 1.0 cm × 0.4 cm × 0.3 cm; (B) 0.8 cm × 0.5 cm × 0.3 cm; (C) lesion from left side measuring 0.9 cm × 0.7 cm × 0.4 cm.

**Figure 4.**
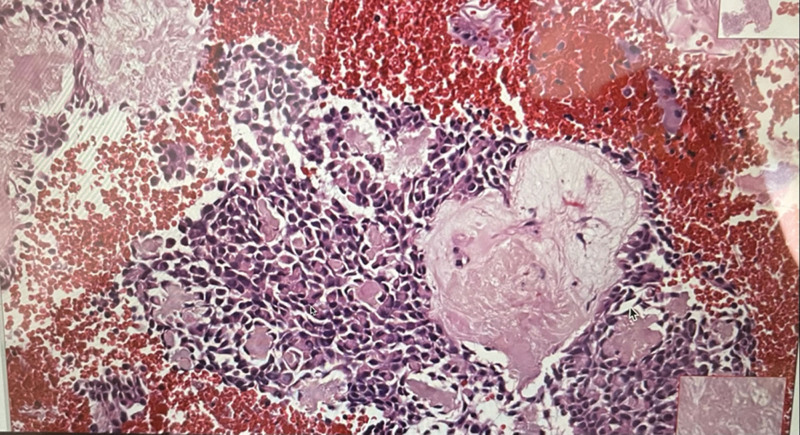
Histological presentation of the lesion (papillary cystic patterns and hyalinized plasmacytoid cells).

The histopathological appearance of the lesion indicated a PA with a well-encapsulated and compressed salivary gland. The tumor cells exhibit a large, round-to-oval shape with a substantial eosinophilic cytoplasm. These cells are observed in both sheets in a glandular pattern. Tumor cells are in a mixture of chondromyxoid material (Fig. 4). Small capsules were present at the periphery, and keratinized cysts were in the cystic space. Papillary cystic patterns were also recorded. The tumor exhibits normal mitosis, and the highly hyalinized plasmacytoid cells were discohesive. A few bone fragments were reported, suggesting the lesion was lying deep in the palate, and surgical excision of the lesion is required.

## 3. Case management

Four weeks after the biopsy, the patient was recalled and readmitted for surgical excision. Informed consent was taken from the patient, and the surgery was performed under anesthesia. During the surgery, a slight saucerization of the underlying bone was noticed, which suggested a pressure effect. An elective surgery was planned to ensure acceptable clearance. The lesion did not penetrate the bone, and the antrum had no mass. The whole tumor mass was removed with precision. The mucosa surrounding the lesion was incised using a surgical blade. A wide surgical dissection was made to remove the entire encapsulated tumor mass, including the mucoperiosteum and eroded bone of the palate. The boundary line was carefully identified in the surrounding healthy tissue. The bleeding was minimal, and hemostasis was obtained using a simple interrupted suture.

Postoperatively, the patient was kept under observation for 2 days and was discharged in stable condition. The patient was given medications and was recalled after 5 days for suture removal. At the time of suture removal, the patient was stable and supportive. The outcome, patient was recalled for follow-up after 1, 3, and 6 months postoperatively, and no sign of reoccurrence was reported. After thorough follow-up over a period of 2 years, no signs of a recurrence were observed.

## 4. Rare cases of PA

PubMed database was searched for rare case reports of Pleomorphic adenoma in English. The MeSH keywords utilized were “Pleomorphic adenoma” OR “epithelial–myoepithelial adenoma” AND “salivary gland” AND “rare case reports” (www.ncbi.nlm.nih.gov/pubmed, accessed on April 16, 2024). The inclusion criteria were the literature published between August 2019 and April 2024, with a well-written presentation of the lesion, treatment, histopathological and clinical findings, and prognosis. The cases, including the floor of the mouth and other external entities, were excluded as it was unclear if the primary site was sublingual or any major salivary gland. This review only focused on the rare cases of PA in the oral cavity.

## 5. Results

The primary search identified 29 articles about the rare cases of PA published in English over the last 5 years (January 2019–April 2024). Of 29 cases, 12 cases satisfied the inclusion criteria. The details of the cases are summarized in Table 1. The included studies varied in patients demographics and tumor locations, the cases ranged from 14-year-old affecting the cleft lip and palate to 72-year-old with a cellular lesion, surgical excision was the primary treatment method used across cases to ensure complete removal and reduce the risk of recurrence, 1 used trans endoscopic mass excision and 1 was local surgery and flap placement. Pain was generally not reported as a prominent symptom, highlighting the often-asymptomatic nature of PA until it grows large enough to cause noticeable symptoms. The cohort included 4 females and 7 males with a median age of 53 years. The distribution of the primary site was as follows: 3 cases with lip, 2 cases each with hard palate and submandibular gland, and 1 case each with tongue, uvula, sublingual gland, cheek, and subglottic gland. Regarding the diagnosis, 7 studies performed the incisional biopsy; 1 performed fine needle aspiration, and 2 performed surgical excision and then reported for pathological examination, and in 2 cases, no specification was given. The tumor size ranged from 1 cm × 1 cm to 12 cm. The disease’s duration ranged from 2 weeks to 48 months, from self-reporting to visiting the hospital. The median postoperative follow-up ranged from 1 month to 36 months. No postoperative complication was reported, and no adjunct radiotherapy and chemotherapy were given to patients.

**Table 1:** Review of clinical and histopathological cases of pleomorphic adenoma.

Sr no.	Investigators/year/country	Presence of pain	Location	Age/gender	Invasion of adjacent tissue	Clinical findings	Histopathological findings	Treatment opted
1	Birigi and Mweya,^[8]^ 2023, Tanzania	No	Submandibular gland	37-year/male	Mandibular bone	Nodular, mobile, firm consistency and non-tender	Not specified	Surgical excision
2	Xiaoru Sun, et al,^[9]^ 2023, China	No	Sub-glucotic	51-year/female	No	Smooth edge, clear boundary, uniform density, and no calcification	Glandular structure and positive for S-100	Surgical excision
3	Liang et al,^[10]^ 2023, China	No	Left side of Tongue	56-year/male	No	Egg-sized swelling on the left side of the tongue extending to the pharynx	Columnar and cuboidal epithelial and myoepithelial cells, and cartilaginous mucus	trans endoscopic mass excision
4	R. R. Skylis et al,^[11]^ 2023, Brazil	No	Tonsil	72-year/female	No	Nodular, firm, and non-tender	A highly cellular lesion with a tendency to cellular monomorphism and scarce stromal component.	Surgical excision
5	Chandra Prakash et al,^[12]^ 2022, India	No	Upper lip	65-year/male	No bony and adjacent tissue involvement	Well-defined, round, hard, non-tender, non-pulsatile, firm consistency	Subepithelial capsulated neoplasm	Surgical excision
6	David sterling et al,^[13]^ 2022, USA	No	Cleft lip and palate	14 years/female	No	Well-defined edges and firm consistency of lesion	Glandular structure	Local excision of lesion and flap placement over cleft palate
7	Vijayakumar et al,^[14]^ 2022, United Kingdom	No	Uvula	43-year/male	No	Not specified	Clear margin and no glandular appearance	Surgical excision
8	AlAmari et al,^[15]^ 2021, Saudi Arabia	No	Nasophyranx	25-years/male	Extending to the soft palate	Smooth edges, round, and no calcification	Plasmacytoid myoepithelial cells	Surgical excision
9	Gupta,^[16]^ 2020, India	No	Hard palate	28-year/male	Extending to the soft palate	Well-defined, round, hard, non-tender, non-pulsatile, firm consistency	Eosinophilic cytoplasm seen in sheets and pseudo glandular pattern	Surgical excision
10	Shah et al,^[17]^ 2020, India	No	Upper lip	68-year/female	No	Firm and non-tender	Epithelial cell arranged in a cord-like cell pattern	Surgical excision
11	Sirohi et al,^[18]^ 2020, India	No	Sublingual salivary gland	64-year/female	No	Firm, well-defined, ovoid, solitary swelling in the left sublingual region	Small clusters, cords, and trabeculae and at places forming micro glandular patterns	Surgical excision
12	Basavaraj Urs, et al,^[19]^ 2019 India	No	Right Cheek region	35-year/male	No	Soft to firm in consistency, mobile, non-fluctuant, non-pulsatile, non-tender	Thick fibrous capsule	Mucosal incision

## 6. Discussion

The prevalence rate of tumors in the salivary gland ranges between 20% and 40%.^[8]^ Smaller affected salivary glands are more likely to demonstrate malignancy. The age group most affected by this condition is in between the fourth and sixth decades of life, with a higher prevalence among females.^[9]^ This condition is primarily observed on the hard and soft palate due to this region’s high concentration of minor salivary glands.^[10]^ PA typically manifests as a gradually enlarging, mainly round or granular shape, painless swelling, with firm consistency.^[10]^ A different embryological etiology can be found for various types of PA. It originates from both epithelial and mesenchymal origins. The mass is delineated from its surroundings by a fibrous capsule. The formation of the capsule occurs due to fibrosis of the surrounding salivary parenchyma, which consists of the tumor and is commonly known as the false capsule.^[11]^ PA is usually a clearly defined and encapsulated tumor.

Clinical diagnosis of this tumor is mainly based on histological examination, history, clinical representation of the lesion, and physical and radiological investigation. The potential differential diagnosis of this condition includes palatal abscess, cysts of odontogenic or nonodontogenic origin, as well as soft tissue tumors such as neurofibroma, fibroma, and neurilemmoma.^[12]^ Palatal abscess can be ruled out through examination, as this condition arises from a non-vital tooth. While examining a tumor, it is possible to rule out the presence of odontogenic and nonodontogenic cysts if the mass does not exhibit a cystic consistency. Myoepithelioma is characterized by spindle-shaped cells and is classified as a benign tumor originating from the epithelial cells of the salivary glands.^[13]^

Radiographically, a computed tomography scan accurately diagnoses the extent of the lesion, as well as bony erosion and invasion. Magnetic resonance imaging scan helps delineate soft tissue spread.^[14]^ Histopathological examination of the specimen exhibits the presence of epithelium and myoepithelial elements arranged in various patterns within a stroma composed of mucopolysaccharides.^[14]^ A pseudo encapsulation is manifested in the lesion because of fibrosis in the adjacent salivary parenchyma, resulting from compression caused by a tumor.^[15]^ The preferred therapeutic approach for PA entails performing a large local excision procedure, including removing the involved periosteum or bone. Simple enucleation of the lesion may result in reoccurrence; hence, it is advisable to perform surgical excision.^[16]^ In the present case, the tumor was present for the last 2 years, which started as a small ulcer and histological examination verified PA with the involvement of small bony fragments; hence, complete enucleation of the lesion was planned.

Palatal reconstruction is contemplated for extensive involvement of palatal bone resulting from surgical removal of highly malignant tumors.^[9]^ In the present case, palate reconstruction was not necessarily due to minimal bony invasion, resulting in the regeneration of palatal mucosa without fistula formation. PAs of the minor glands have a small probability for recurrence (a recurrence rate of 2–44%, although primarily of the parotid gland), in contrast to surgical exposure of the tumor or its capsule, which carries the risk of spillage and raises the chance of recurrences.^[7]^ Recurrent PAs frequently give rise to multiple nodules in various locations, such as the remaining salivary gland, peri parotid tissues, dermis tissue, and scar tissue, even several years or decades after the initial surgical treatment.^[17]^ Inadequate or minimal surgical treatments were identified as the primary factor responsible for the recurrence. The common surgical complications include pseudopodia, capsular penetration, and tumor rupture. In several cases, distant metastasis was also reported.^[18–20]^

The present case report and the report on rare cases demonstrate that PA arises in the form of small ulcers and, if left untreated, increases in size over time. However, no reported cases have observed the metastasis and recurrence of the lesion. Studies have reported that around 50% of minor salivary gland tumors are malignant, with the prevalence of adenoid cystic carcinoma.^[19,20]^ PA arising from the palate, buccal mucosa, and lip lacks encapsulation, resulting in enlargement of host tissue; hence, wide excision of the lesion is required even if biopsy suggests a benign lesion. Hence, deep excision and the removal of bone fragments were performed in the present case.

Studies have reported that PA are radio resistant, hence radiotherapy is contraindicated.^[18,19]^ Malignant transformation, though rare, has been reported in about 3% to 5% of cases. The risk of recurrence of PA is generally associated with inadequate surgical procedures and involving the risk of pseudopodia, capsular penetration, and tumor rupture.

## 7. Conclusion

Clinical examination and histopathological findings are important to diagnose PA. PA usually involves extensive squamous and mucous metaplasia, confusing it with malignant disorders such as mucoepidermoid and adenoid cystic carcinoma. The absence of metastasizing cells suggests definitive PA. Complete surgical excision of PA is required due to the metastasizing nature of the lesion. The reviews reported, and management of the case in this report suggest that wide excision with a negative margin is optimal to manage PA. Regular follow-up and histopathological investigation should be performed to reduce the risk of recurrence.

### 7.1. Limitation and challenging

Limitations present in this review is inclusion of rare cases reported in last 5 years and only published in English language.

Single case, this focus on individual patient, made it difficult to generalize the finding.

The follow-up phase posed significant challenges; however, we achieved long term follow-up period that enabled us to effectively manage and monitor the case.

## Author contributions

**Conceptualization:** Lujain AlSahman, Roba AlSahman.

**Investigation:** Lujain AlSahman, Osama Alghamdi, Roba AlSahman.

**Methodology:** Lujain AlSahman, Roba AlSahman.

**Project administration:** Lujain AlSahman.

**Resources:** Lujain AlSahman, Roba AlSahman.

**Supervision:** Osama Alghamdi, Hamad Albagieh, Ali AlRefai.

**Validation:** Lujain AlSahman.

**Visualization:** Lujain AlSahman, Shatha Alnafea.

**Writing – original draft:** Lujain AlSahman, Roba AlSahman.

**Writing – review & editing:** Lujain AlSahman, Roba AlSahman.
